# Life-history and reproductive traits of a key coral reef fishery species: the longnose emperor (*Lethrinus olivaceus*) in Palau

**DOI:** 10.7717/peerj.21247

**Published:** 2026-05-07

**Authors:** Christina Muller Karanassos, Brett M. Taylor, Alexander Filous, Steve Lindfield, Nils C. Krueck, Barrett Wolfe, Alyssa Marshell

**Affiliations:** 1 Palau International Coral Reef Center, Koror, Palau; 2 Institute for Marine and Antarctic Studies, University of Tasmania, Hobart, Tasmania, Australia; 3 Center for Island Sustainability and Sea Grant, Marine Laboratory, University of Guam, Guam, United States of America; 4 The Nature Conservancy, Koror, Palau; 5 Coral Reef Research Foundation, Koror, Palau

**Keywords:** Age and growth, Size at maturity, Lethrinidae, Small-scale fisheries, Indo-Pacific, Fisheries management, Data-poor fisheries

## Abstract

Accurate life-history information is essential for conducting data-limited stock assessments of coral reef fishes, yet this information is often lacking in small-scale fisheries. For the commercially important longnose emperor, *Lethrinus olivaceus*, such information is scarce in Micronesia, limiting the ability to manage this species effectively. To fill this gap, age, growth, and reproductive parameters were estimated for *L. olivaceus* in Palau using samples collected at fish markets, supermarkets, independent fishers, and targeted sampling in 2017–2019 and 2022–2025 (total *n* = 528). Information on growth, lifespan, reproduction, and mortality was obtained through analysis of sagittal otoliths and gonad samples. The sex-combined von Bertalanffy growth parameters for this species were estimated to be *L*_∞_ = 57.2 cm,* K* = 0.387 year^−1^, and *t*_0_ = −0.324 years (unconstrained) and *L*_∞_ = 56.4 cm, and *K* = 0.443 year^−1^ (constrained to *t*_0_ = 0). The maximum observed age was 14 years. Longnose emperors were found to spawn in many months of the year including March to June, August, September, November, and December. There was also evidence of functional protogynous hermaphroditism, with several gonad samples containing both female and male reproductive tissues. Furthermore, males were larger (mean length = 53.5 cm) and older (mean age = 7.4 years) than females (mean length = 45.8 cm and mean age = 4.3 years), with no immature males observed. Females were estimated to reach maturity at 43.2 cm and 3.5 years, and sex change to males occurred at 55.1 cm and 7.1 years. Total mortality for both time periods combined was estimated to be 0.438 year^−1^ and mean natural mortality was estimated to be 0.358 year^−1^. When compared to other locations at higher latitudes, including Australia, Japan, and French Polynesia, longnose emperors in Palau had faster growth rates, shorter lifespans, and reached smaller sizes. The high diversity of life-history traits among locations highlights the importance of collecting locally-derived information. These findings provide comprehensive life-history estimates for *L. olivaceus* in Palau, which can be used to improve data-poor assessments and management of this species in the future.

## Introduction

Emperors (family Lethrinidae) are widespread throughout the Indo-Pacific region and are important in both commercial and subsistence fisheries (Carpenter & Allen, 1989). For this reason there have been numerous studies examining the life-history traits of lethrinid species across their range (*e.g.*, Ebisawa & Ozawa, 2009; Marriott et al., 2010; Taylor et al., 2018; Pardee & Wiley, 2022; Trianni, Demartini & Taylor, 2023; Lin et al., 2024; Chandravanshi et al., 2025). Many species are considered to be protogynous hermaphrodites, changing sex from female to male during their lifetime (Young & Martin, 1982). Several emperors have also been reported to form spawning aggregations in distinct seasons relating to specific lunar phases (*e.g.*, Hamilton, 2005; Taylor & Mills, 2013; Babcock, Pillans & Rochester, 2017). These reproductive strategies may make lethrinid species more vulnerable to population decline resulting from overharvest since the removal of larger fish can lead to a skewed sex ratio (Alonzo & Mangel, 2004), and spawning aggregations are often targeted by fishers who know exactly where and when to find them (Domeier, 2012).

The longnose emperor (*Lethrinus olivaceus*) is the largest species in the emperor family, reaching a maximum reported size of 100 cm total length (TL) (Allen et al., 2003). This species is known to form spawning aggregations that are commonly targeted by fishers (Filous et al., 2022). However, studies of its life history remain scarce, with published examples including those from Australia (Currey et al., 2013), Japan (Shimose, 2021), and French Polynesia (Filous et al., 2022). These studies indicate that *L. olivaceus* has a moderate lifespan, reaching a maximum age of 14 to 22 years, with an asymptotic length (*L*_∞_) ranging from 66 to 69.9 cm fork length (FL) (Currey et al., 2013; Shimose, 2021; Filous et al., 2022). In French Polynesia, females were found to mature at around 3 years (38 cm TL; 34.4 cm FL) with sex change occurring at 4 years (45 cm TL; 40.9 cm FL) (Filous et al., 2022).

Life-history traits such as size, growth, age structure, and maturation are known to vary across temperature gradients and geographic locations (Atkinson, 1994; Clarke, 2003; Forster, Hirst & Atkinson, 2012; Taylor et al., 2021; Lowe et al., 2021). Generally, fish at higher latitudes grow more slowly, attain larger maximum sizes, live longer, and mature later (Trip et al., 2014; Álvarez-Noriega et al., 2023). In comparison to other locations where *L. olivaceus* has been studied, Palau is located at a lower latitude, has higher seawater temperatures and lower seasonality year-round. Based on these conditions, we hypothesized that *L. olivaceus* in Palau would exhibit a faster life-history strategy, characterized by faster growth, smaller maximum size, shorter lifespan, and earlier maturation.

*L. olivaceus* is a key species in Palau’s multispecies reef fishery, accounting for approximately 4% of total landings in 2023–2024 (Muller Karanassos et al., in press). This species was recently confirmed to form spawning aggregations around the new moon each month in Palau (Muller Karanassos et al., 2026). These spawning aggregations are targeted by fishers, a behaviour which may contribute to declining catches (Johannes, 1981; Sadovy, 2007). A recent length-based stock assessment found that *L. olivaceus* has likely been depleted to a relative spawning potential ratio of only 29%, and the associated ratio of long-term fishing mortality to natural mortality (*F/M*) of 1.73 indicates this species is vulnerable to further depletion in Palau (Muller Karanassos et al., in press). However, data-limited stock assessments are highly sensitive to life-history parameter inputs (Hordyk et al., 2014; Medeiros-Leal et al., 2023) and, where possible, locally-derived information (particularly size at maturity—*L*_50_) can fundamentally improve stock assessments to accurately reflect the state of the fishery. Although size at maturity has previously been estimated for *L. olivaceus* in Palau, including using macroscopic methods in the northern reefs (40.5 cm—Prince et al. (2015) and 40.9 cm—Prince (2016)) and histological methods in the south (42.3 cm) (Prince et al., 2022), age and growth parameters have not been estimated for this species in Palau.

Currently, there are no formal management measures in place for *L. olivaceus* in Palau. However, a proposed coastal fisheries management plan for the state of Koror includes a minimum size limit of 18 inches (45.7 cm) and the protection of a spawning aggregation site. The management plan was developed in 2021 but has not yet been implemented. Effective management of *L. olivaceus* is complicated by its sex-changing biology, which may require the protection of larger males through the implementation of maximum size limits (Gwinn et al., 2015). However, a lack of locally-derived life history information—including age, growth, and reproductive parameters—limits the ability to evaluate these management measures and ensure sustainable harvests. Therefore, the main aim of this study was to estimate age and growth parameters for *L. olivaceus* in Palau and build on existing reproductive information for the species, including characterization of spawning seasonality and an updated estimate of size at maturity. This information will improve the accuracy of future stock assessments and provide a scientific basis for management decisions, including the evaluation of appropriate minimum and maximum size limits for this important reef fishery species.

## Materials & Methods

### Sample collection

To characterize the demography of *L. olivaceus* in Palau, specimens were purchased opportunistically from the main fish market in Koror state (JR5 fish market), supermarkets, and independent fishers from November 2022 to February 2025 (*n* = 325). Fishing gear used to capture individual fish was not recorded for the market-sampled specimens, however in Palau, *L. olivaceus* are primarily caught using bottom hook-and-line fishing and night spearfishing. Additional targeted fishing was conducted at spawning aggregation sites for under-represented size classes of larger fish (*n* = 74) using bottom fishing. The different fishing methods used may exhibit some size selectivity, which could influence the size composition of samples and therefore the estimation of growth parameters. Fish sampling activities were conducted in accordance with University of Tasmania Animal Ethics Committee Permit No. 28012. Captured fish were euthanised by using blunt force trauma to the head followed by exsanguination (Robb et al., 2000) and then placed in a seawater ice slurry until dead (Blessing, Marshall & Balcombe, 2010). Fish were kept on ice until processing at the Palau International Coral Reef Center (PICRC) lab, where they were measured to the nearest 0.1 cm (FL and TL) and weighed to the nearest 0.1 g (total weight). Additional *L. olivaceus* samples had previously been collected by the Coral Reef Research Foundation (CRRF), primarily from the JR5 fish market, from 2017 to 2019 (*n* = 129). Gonad samples had already been processed and analysed for a previous project by CRRF (Lindfield et al., 2020) whereas otolith samples were processed and analysed specifically for the current study. All statistical analyses were conducted using R version 4.4.0 (R Core Team, 2024).

### Morphometric relationships

Morphometric relationships were examined to allow conversion between length measurements and to describe the length-weight relationship for *L. olivaceus* in Palau. A linear least squares regression analysis was used to examine the relationship between fork length and total length, using the equation:

\begin{eqnarray*}TL=a+bFL \end{eqnarray*}

where *TL* is the total length (cm), *FL* is the fork length (cm), *a* is the intercept, and *b* is the slope. Not all fork length measurements had an associated total length measurement (*n* = 448). The length-weight relationship was modelled using the equation:

\begin{eqnarray*}W=aF{L}^{b} \end{eqnarray*}

where *W* is the total weight of the fish (g), *FL* is the fork length (cm), and *a* and *b* are constants. Parameters *a* and *b* were estimated directly from the raw data using nonlinear least-squares regression. 95% confidence intervals were calculated using a bootstrap approach (*R* = 1,000 resamples).

### Age and growth

Sagittal otoliths of 511 *L. olivaceus* were used for age determination. Otoliths were extracted from the fish, cleaned, and stored dry before processing following methods in Pardee et al. (2020). Otoliths were weighed to the nearest 0.0001 g, the core was marked with a fine pencil, and the otoliths were mounted onto glass slides using Crystalbond 509 adhesive with the core positioned just inside the edge of the slide. Otoliths were then sectioned transversely using a Ted Pella XP 20 grinder mounted with 400–1,200-grit discs to produce sections ∼0.2–0.3 mm thick, including the core region. Otolith sections were then viewed under a stereo microscope with transmitted and reflected light, and the number of annuli were counted independently by three readers. The age was determined when at least two counts agreed, and if three counts differed by one annulus, the middle count was used to determine age (Pardee et al., 2020). Where readings differed by more than one annulus, the otolith was re-read and if this age agreed with one of the original readings, then this was used as the final age. Previous studies have confirmed that translucent and opaque increments in otoliths of *L. olivaceus* are deposited annually (Currey et al., 2013; Shimose, 2021; Filous et al., 2022). Otolith weight was plotted against age using a linear regression to measure the capacity of otolith weight as a predictor of age.

Sex-specific growth analyses were not conducted because *L. olivaceus* is thought to be a functional protogynous hermaphrodite (Shimose, 2021), with individuals maturing first as females and transitioning to males at larger sizes and older ages. Consequently, males are absent from younger age classes and represent individuals that have already undergone female growth, making sex-specific growth comparisons inappropriate. Therefore sex-combined length-at-age data were fitted to the von Bertalanffy growth function (VBGF) and growth parameters were estimated using least squares non-linear regression:

\begin{eqnarray*}{L}_{t}={L}_{\infty }[1-{e}^{-K \left( t-{t}_{0} \right) }] \end{eqnarray*}

where *L*_*t*_ is the mean fork length (cm) at age *t* (years), *L*_∞_ is the mean asymptotic fork length (cm) representing the theoretical maximum size approached by individuals, *K* describes the rate at which individuals approach *L*_∞_, and *t*_0_ is the theoretical age at which fork length equals zero. Due to a lack of small individuals sampled, and for comparison to other studies, VBGF growth parameters were also estimated with *t*_0_ constrained to zero (*t*_0_ = 0).

### Maturity and reproduction

Gonads from each *L. olivaceus* were removed and weighed to the nearest 0.01 g (gonad weight). The gonadosomatic index (GSI) was calculated as the ratio of gonad-to-gonad-free body weight for each mature fish using the equation:

\begin{eqnarray*}GSI= \left( \frac{Gonad~weight}{Total~weight-Gonad~weight} \right) \ast 100 \end{eqnarray*}

Gonads from all 528 *L. olivaceus* specimens were used for histological analysis. Samples were collected and processed as previously described in Muller Karanassos et al. (2026). Briefly, gonadal tissue (∼4 mm thick) was excised from the mid-lobe region, fixed in 10% neutral buffered formalin, and sent to the Histopathology Core Facility at the University of Hawaii for processing. Following standard protocols described by Sullivan-Brown, Bisher & Burdine (2011), gonad sections were embedded with paraffin wax, sectioned transversely at 6 µm, stained with haematoxylin and eosin, and mounted on glass slides. Samples were analysed using a compound microscope to determine sex and maturity stages based on standardised terminology developed by Brown-Peterson et al. (2011) and adapted by Taylor et al. (2018) and Prince et al. (2022) for Indo-Pacific reef fish. Female reproductive phases were determined based on the most advanced oocyte developmental stage present in the ovary (*e.g.*, primary growth, cortical alveoli, vitellogenic, or hydrated oocytes) and the presence of post-ovulatory follicles or atretic oocytes. Females classified as “Immature” or “Developing” were categorised as immature, whereas females classified as “Spawning capable”, “Actively spawning”, “Regressing”, or “Regenerating” were categorised as mature. Males were classified as mature based on the presence of tailed spermatozoa in the lobules of testes. To ensure consistency in stage assignment, slides with ambiguous or uncertain features were re-examined by the primary reader, and a subset of slides was independently reviewed by a second reader.

Since *L. olivaceus* is thought to be a functional protogynous hermaphrodite (Shimose, 2021), size and age at maturity were estimated for females only. Size at 50% and 95% maturity (*L*_50_ and *L*_95_) was modelled using a logistic regression with fork length as the explanatory variable and maturity stage (0 = immature, 1 = mature) as the response. Age at 50% and 95% maturity (*A*_50_ and *A*_95_) was similarly estimated using a logistic regression with age as the explanatory variable and individual maturity (0 = immature, 1 = mature) as the response. Size at sex change, defined as the size at which 50% (*L*_50_*SC*) and 95% (*L*_95_*SC*) of individuals had transitioned to males, and age at 50% and 95% sex change (*A*_50_*SC* and *A*_95_*SC*), were estimated using logistic regression with sex as a binary response (0 = female, 1 = male). Confidence intervals for all estimates were calculated using bootstrap resampling with 1,000 iterations.

To examine spawning season, the proportion of females in each spawning state (actively spawning, spawning capable, regressing, regenerating, developing, and immature) was plotted across months.

### Mortality

For total mortality (*Z*) estimates, samples collected during targeted fishing activities at spawning aggregation sites (*n* = 74) were excluded from analysis due to sampling bias of larger size classes. Total mortality was estimated for *L. olivaceus* for both time periods (2017–2019 and 2022–2025) combined (*n* = 440). A linearized catch curve developed by Beverton & Holt (1957) was applied using the equation:

\begin{eqnarray*}\ln \nolimits \left( {C}_{t} \right) =\ln \nolimits \left( {N}_{0} \right) -Zt \end{eqnarray*}

where *C*_*t*_ is the catch at age *t*, *N*_0_ is the recruitment into the cohort, and *Z* is the instantaneous total mortality rate, with *t* representing age. The estimation of *Z* was based on the portion of the catch curve where the natural logarithm of fish abundance decreases with age—that is, the declining section following the peak age class. Age groups with very few individuals (one or fewer) were excluded from the analysis to reduce bias caused by sparse data, following the approach recommended by Dunn, Francis & Doonan (2002).

Natural mortality (*M*) was estimated for *L. olivaceus* using three empirical approaches for comparison, based on the maximum age observed in the population (*t*_*max*_):

1. Hoenig (1983):
\begin{eqnarray*}\ln \nolimits \left( M \right) =1.46-1.01ln({t}_{\mathrm{max}}) \end{eqnarray*}
2. Then et al. (2015):
\begin{eqnarray*}\ln \nolimits \left( M \right) =1.717-1.01ln({t}_{\mathrm{max}}) \end{eqnarray*}
3. Hamel & Cope (2022):
\begin{eqnarray*}M= \frac{5.4}{{t}_{\mathrm{max}}} \end{eqnarray*}

For the first two methods, natural mortality was calculated by back-transforming the logarithmic values. These complementary estimators were applied to provide a more robust characterization of natural mortality in the study population.

Fishing mortality (*F*) was estimated as the difference between total mortality (*Z*) and natural mortality (*M*):

\begin{eqnarray*}F=Z-M \end{eqnarray*}

To assess fishing pressure, the ratio between fishing mortality to natural mortality (*F/M*) was also estimated.

### Assumptions of models

For analyses using linear regression, including morphometric relationships and the linearized catch curve, assumptions were evaluated through visual inspection of diagnostic plots, including residuals *versus* fitted values and normal Q–Q plots, to assess homoscedasticity and approximate normality of residuals. For nonlinear models, including the von Bertalanffy growth function and the length–weight relationship, model fit was assessed by examining residual plots to ensure no obvious patterns or deviations from expected trends. No major violations of model assumptions were observed.

## Results

### Size structure and morphometric relationships

**Figure 1:**
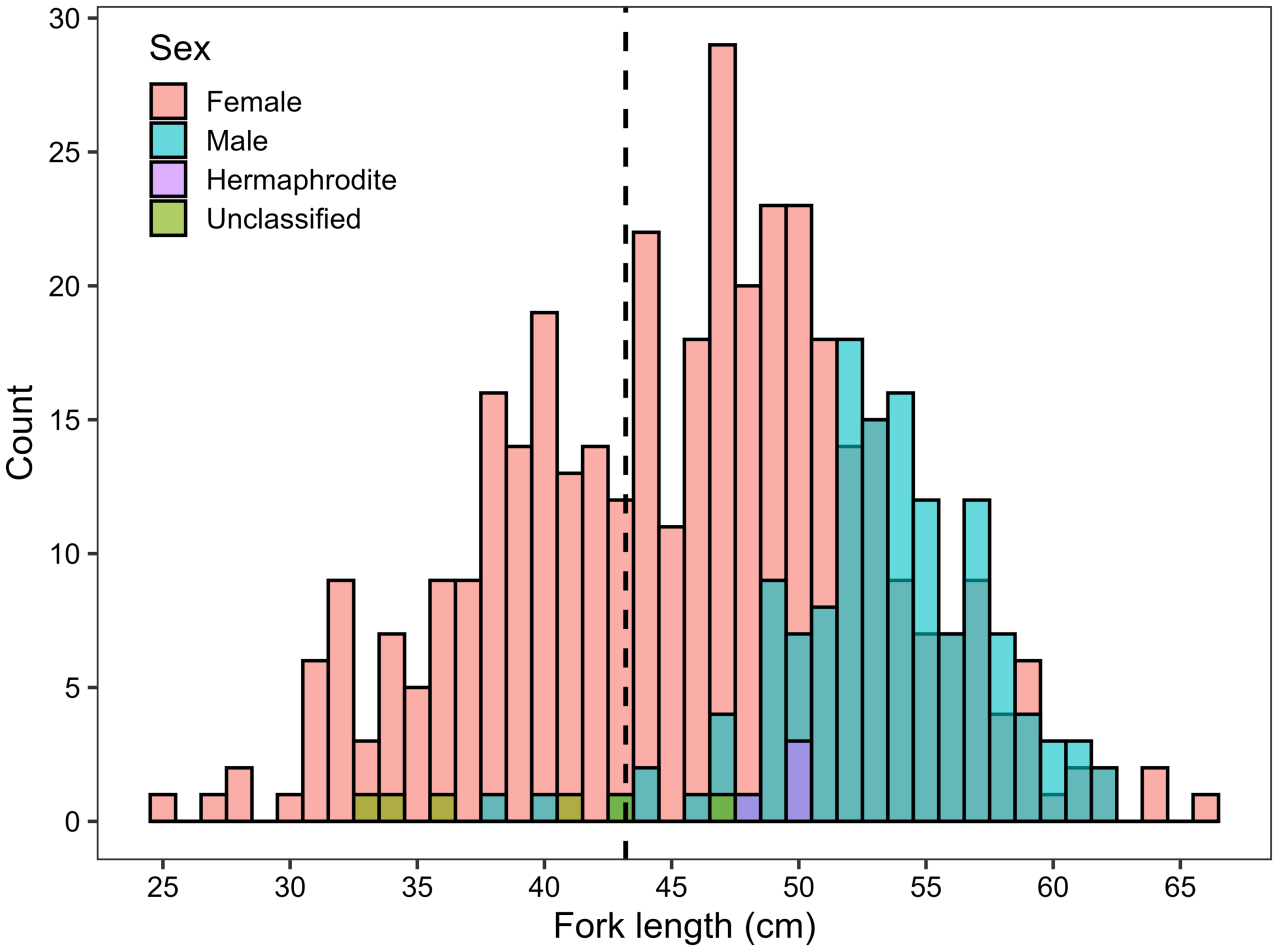
Length-frequency histogram of *L. olivaceus* sampled in Palau in 2017–2019 and 2022-2025 with sex shown by different colours. The black dashed line indicates the female size at 50% maturity (43.2 cm) estimated in this study.

Of the 528 *L. olivaceus* that were sampled in Palau in 2017–2019 and 2022–2025, 384 were female, 134 were male, four were hermaphrodites, and six were unclassified. The modal length for males was greater than the modal length for females. The fork length of females ranged from 25.4 to 65.7 cm, with a mean of 45.8 cm ± 0.375 cm (SEM), and the fork length of males ranged from 37.6 to 62.3 cm, with a mean of 53.5 cm ± 0.356 cm (Fig. 1).

Fork length was strongly related to total length, with a linear relationship indicating proportional growth across the observed size range (*R*^2^ = 0.987) (Table S1 and Fig. S1A). The length-weight relationship (FL cm to grams) followed a power function, with body mass scaling close to isometrically with fork length (Table S1 and Fig. S1B).

### Age and growth

**Figure 2:**
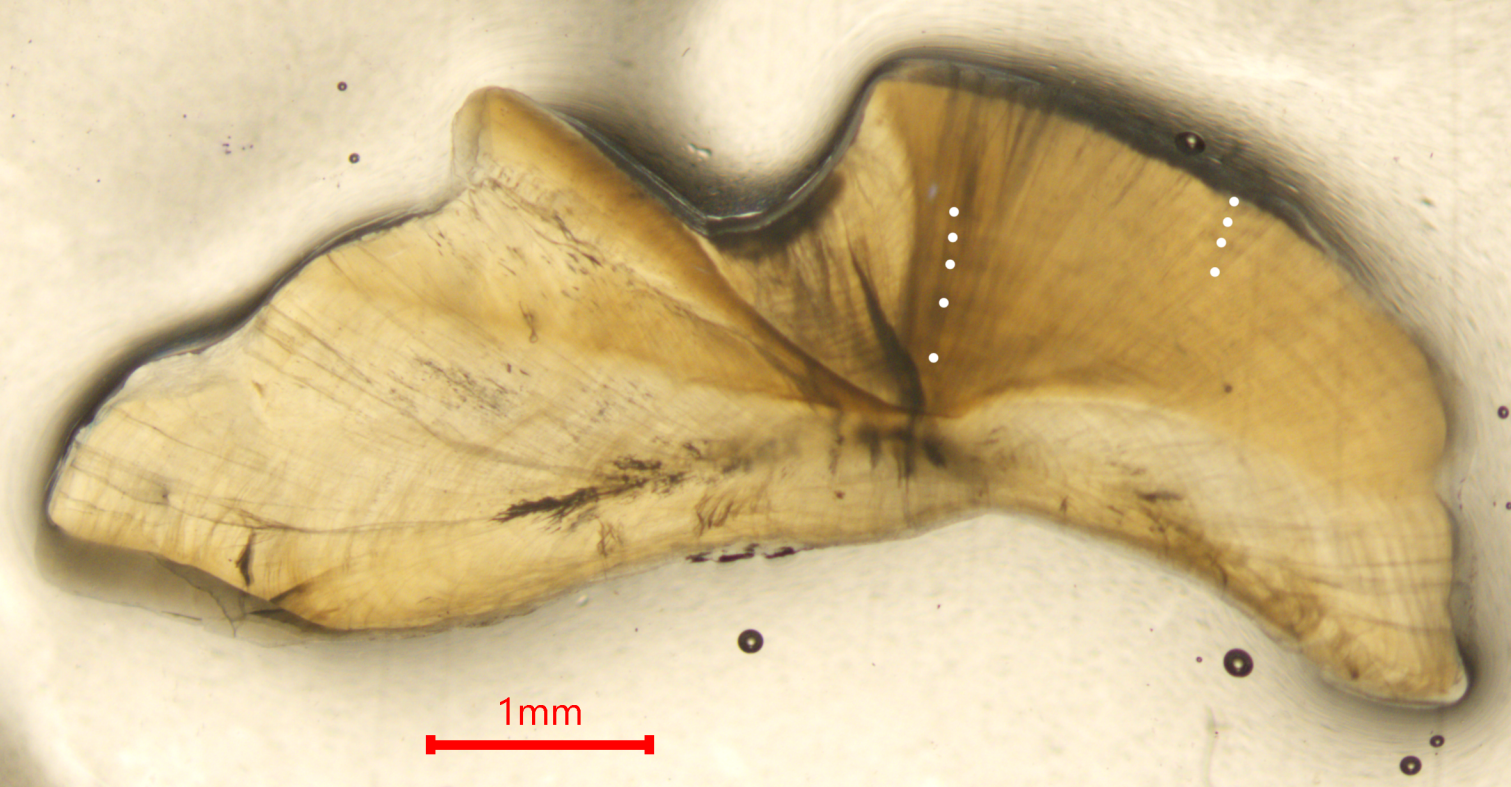
Photomicrograph of a sectioned otolith from a 62 cm (FL) nine-year-old female *L. olivaceus* with annuli shown by white points.

**Figure 3:**
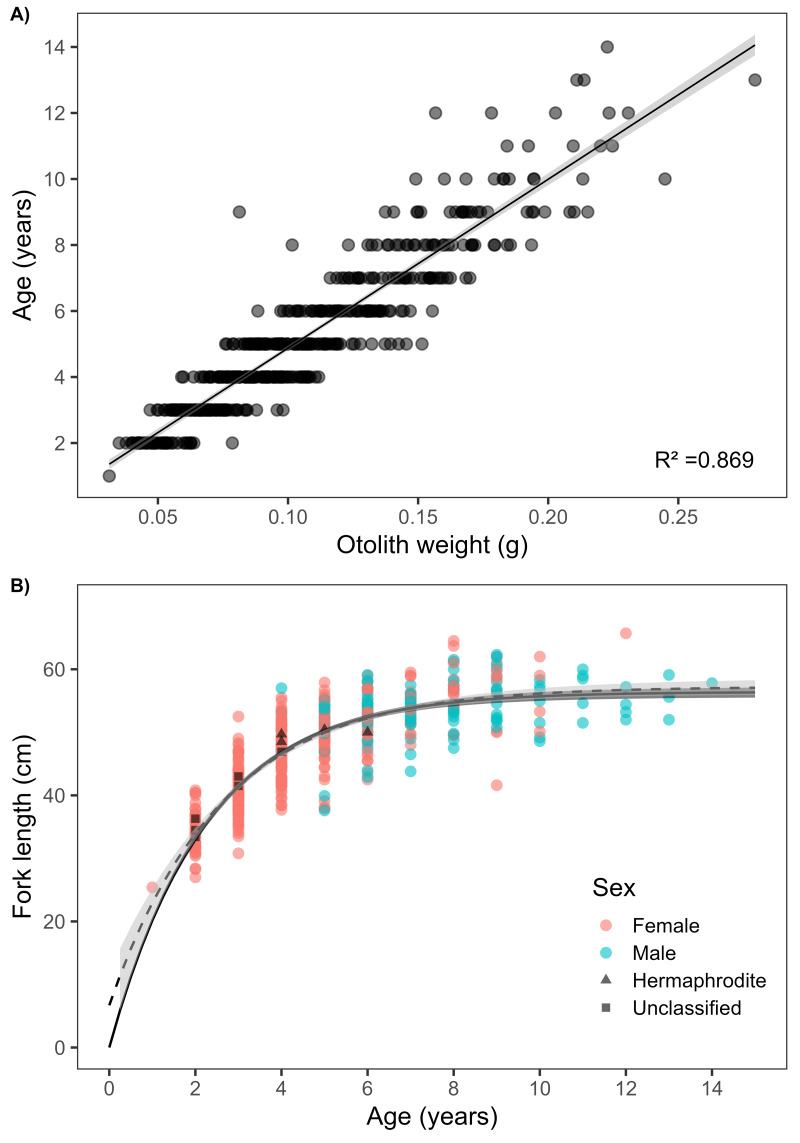
Sex-combined relationship between *L. olivaceus* otolith age and weight (A) and unconstrained (dashed line) and constrained (solid line) von Bertalanffy growth curves (B). Lines represent the best-fit curves for both sexes combined (*n* = 511) with 95% confidence intervals shown by light grey ribbon (unconstrained) and dark grey ribbon (constrained).

**Figure 4:**
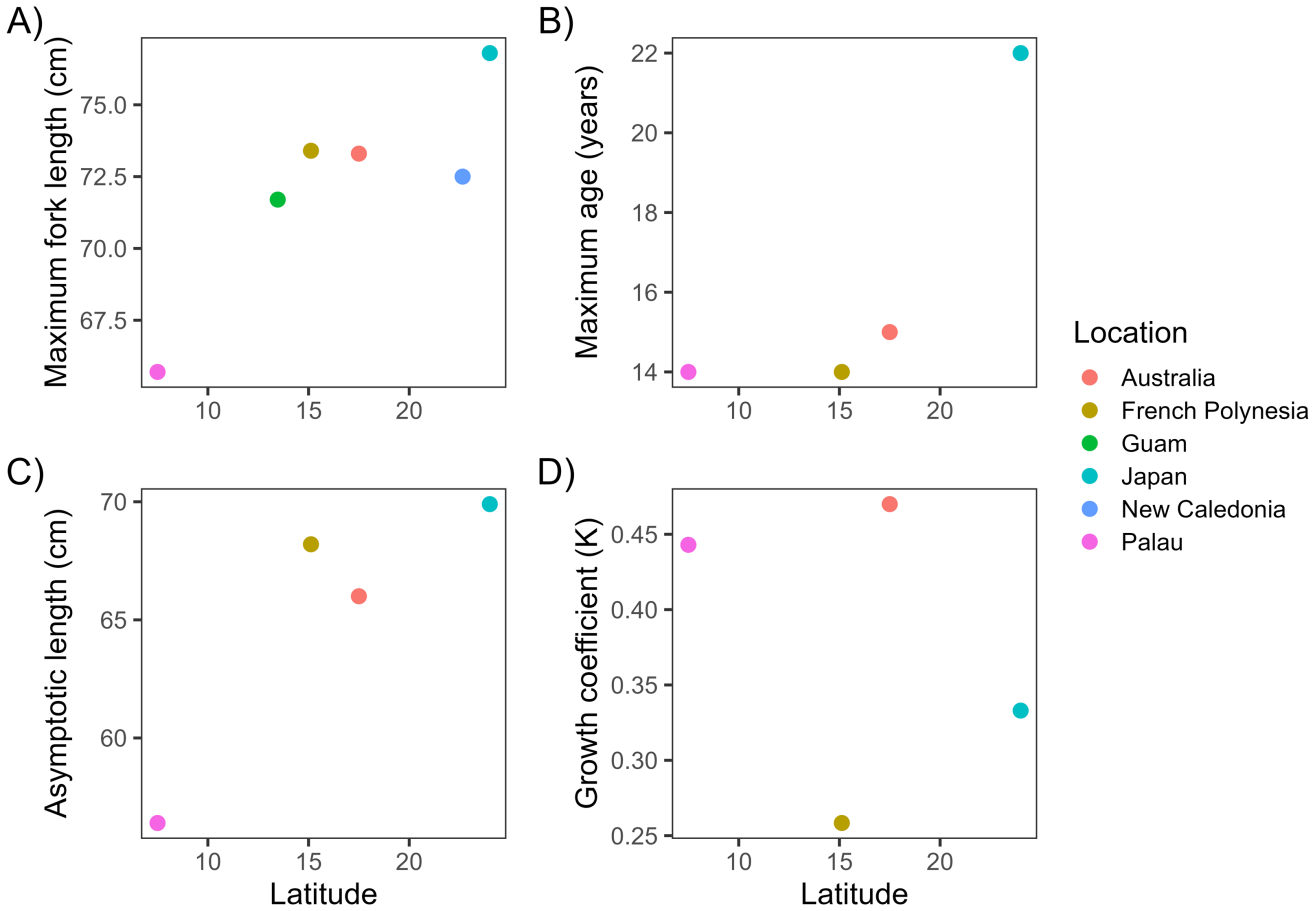
Comparison of life-history parameters for *L. olivaceus* across latitudes (°), including maximum fork length (A), maximum age (B), asymptotic length (C), and growth coefficient K (D). *L*_∞_ and *K* values were all estimated with the VBGF curve constrained to *t*_0_ = 0.

Otoliths displayed clearly defined annuli, consistent with those observed in previously studied emperor species (Fig. 2). There was a strong linear relationship between otolith weight and age (*R*^2^ = 0.869) (Fig. 3A). Of the 511 *L. olivaceus* that were aged, the minimum age was 1 year, and the maximum age was 14 years. Females ranged in age from 1 to 12 years (mean = 4.3 years ± 1.8 SD), and males ranged in age from 4 to 14 years (mean = 7.4 years ±  2.2 SD). The unconstrained von Bertalanffy growth parameters, including 95% confidence intervals, were estimated to be *L*_∞_ = 57.2 (56.0–58.5) cm, *K* = 0.387 (0.335–0.445) year^−1^, and *t*_0_ = −0.324 (−0.695 to −0.006) years for both sexes combined. The constrained von Bertalanffy growth parameters were estimated to be *L*_∞_ = 56.4 (55.5–57.2) cm and *K* = 0.443 (0.423–0.465) year^−1^ (Fig. 3B and Table 1). The maximum size for *L. olivaceus* from this study was 65.7 cm FL. Compared to other study sites, *L. olivaceus* in Palau were generally smaller (Fig. 4A and Table S2), had shorter lifespans (Fig. 4B), reached lower asymptotic lengths (Fig. 4C), and had a faster growth rate (Fig. 4D).

**Table 1:** Summary of life-history parameter estimates for *L. olivaceus* in Palau including data from 2017–2019 and 2022–2025.

	Combined	Females	Males
*A*_*min*_ (years)	1	1	4
*A*_*max*_ (years)	14	12	14
*A*_50_ (years)	–	3.5 (3.4–3.7)	–
*A*_95_ (years)	–	5.4 (4.9–5.9)	–
*A*_50_*SC* (years)	–	–	7.1 (6.7–7.7)
*A*_95_*SC* (years)	–	–	11.3 (10.1–12.7)
*L*_50_ (cm)	–	43.2 (42.5–44.0)	–
*L*_95_ (cm)	–	50.2 (48.7–51.7)	–
*L*_50_*SC* (cm)	–	–	55.1 (53.8–56.9)
*L*_95_*SC* (cm)	–	–	69.3 (65.2–74.4)
*L*_∞_ (cm) unconstrained	57.2 (56.0–58.5)	–	–
*K* (year^−1^) unconstrained	0.387 (0.335–0.445)	–	–
*t*_0_ (years) unconstrained	−0.324 (−0.695 to −0.006)	–	–
*L*_∞_ (cm) constrained	56.4 (55.5–57.2)	–	–
*K* (year^−1^) constrained	0.443 (0.423–0.465)	–	–
*Z* (year^−1^)	0.438 (0.335–0.541)	–	–
*M*_*Then*_ (year^−1^)	0.387	–	–
*M*_*Hoenig*_ (year^−1^)	0.300	–	–
*M*_*HamelCope*_ (year^−1^)	0.386	–	–
Mean *M* (year^−1^)	0.358 ± 0.05	–	–
*F* (year^−1^)	0.080	–	–
*F/M*	0.220	–	–

**Notes.**

*A*_*min*_ minimum age *A*_*max*_ maximum age *A*_50_ age at 50% maturity *A*_95_ age at 95% maturity *A*_50_*SC* age at 50% sex change *A*_95_*SC* age at 95% sex change *L*_50_ size at 50% maturity *L*_95_ size at 95% maturity *L*_50_*SC* size at 50% sex change *L*_95_*SC* size at 95% sex change *L*_∞_ mean asymptotic length K von Bertalanffy growth coefficient *t*_0_ age at which fork length equals zero Z total mortality M natural mortality (Then et al., 2015; Hoenig, 1983; Hamel & Cope, 2022) F fishing mortality F/M ratio of fishing mortality to natural mortality

Length estimates are in fork length (cm), ages are in years, and 95% confidence intervals are included in brackets.

### Maturity and reproduction

**Figure 5:**
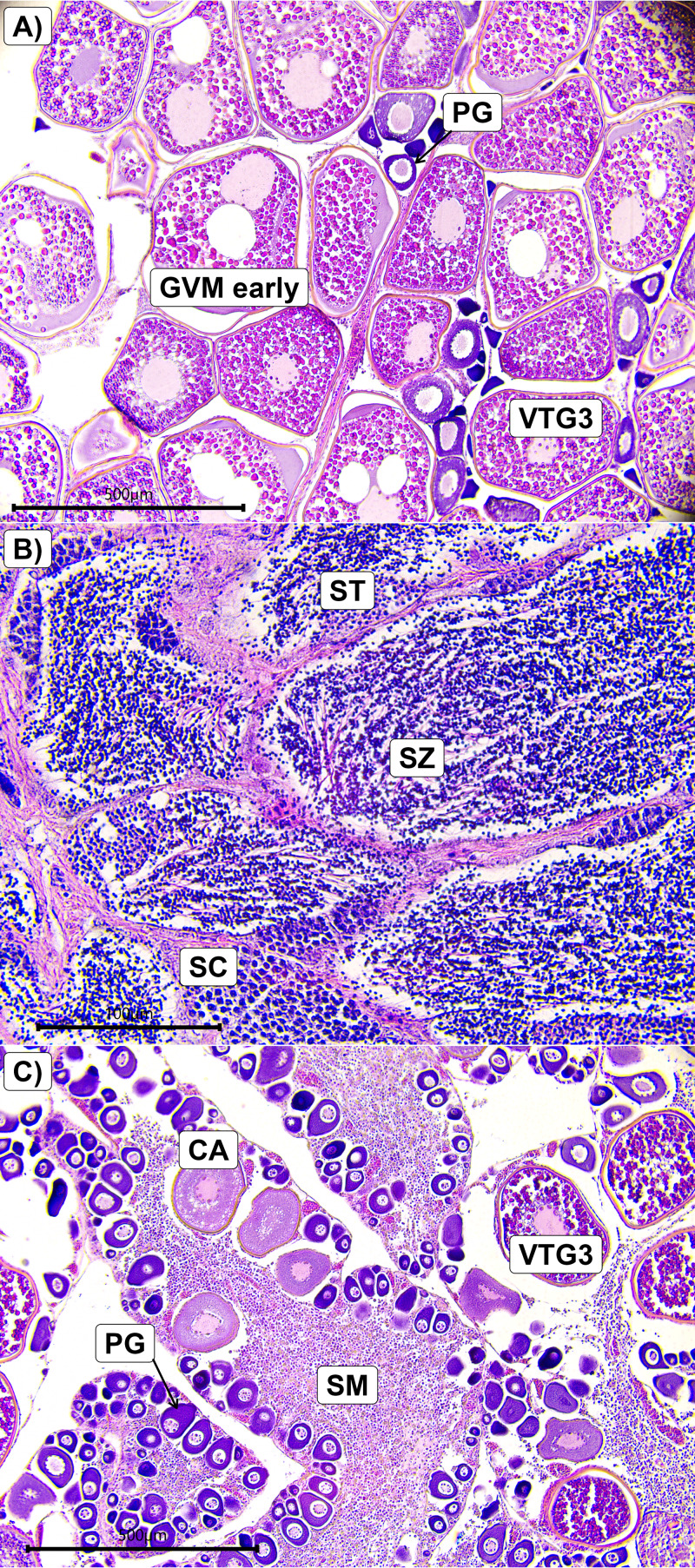
Photomicrographs of histological slides from *L. olivaceus* showing a spawning capable female (A), a spawning capable male (B), and a hermaphrodite (C). Codes represent the following: PG, primary growth; CA, cortical alveolar; VTG3, tertiary vitellogenic; GVM early, early germinal vesicle migration; SZ, spermatozoa; SC, spermatocytes; ST, spermatids; SM, spermatogenic material. The scale bar is 500 µm in A and C, and 100 µm in B.

Of the 134 males sampled, 115 were classified as spawning capable (Fig. 5A), 11 were regressing, four were regenerating, and four were developing. No males were classified as immature. Of the 384 females sampled, 41 were classified as actively spawning (Fig. 5B), 149 were spawning capable, five were regressing, 45 were regenerating, 20 were developing, and 124 were immature. The six unclassified fish were immature. Of the four *L. olivaceus* that were classified as hermaphrodites, three had mature female tissue (atresia and/or VTG3 oocytes) whereas one only had immature oocytes, in addition to developing spermatogenic material (Fig. 5C). All four hermaphrodites were above the estimated female size at maturity (Fig. 1).

**Figure 6:**
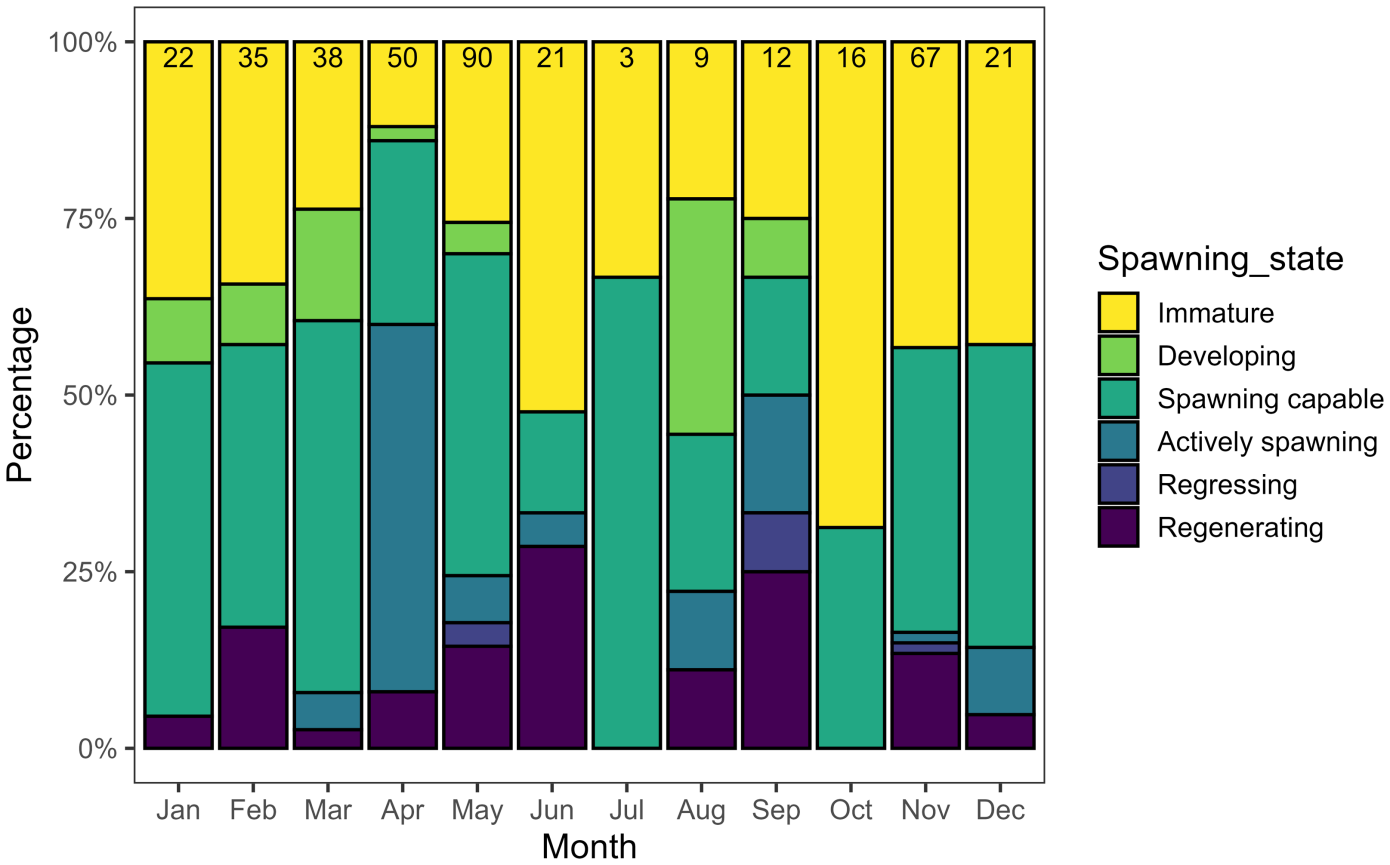
Spawning state of female *L. olivaceus* across months of the year sampled in Palau in 2017–2019 and 2022–2025 (*n* = 384). Numbers above bars indicate the number of samples collected in each month.

Although sample sizes varied across months and lunar phases, reproductively active females were observed in all months of the year, with evidence of active spawning seen from March to June, August, September, November, and December (Fig. 6). GSI of mature fish was highest on the days around the new moon (Fig. S2). For females, a maximum GSI of 7.8 was recorded in September, with the highest mean GSI occurring in April (2.92 ±  0.23). For males, a maximum GSI of 2.5 was recorded in May, with the highest mean GSI occurring in March (1.04 ± 0.20).

**Figure 7:**
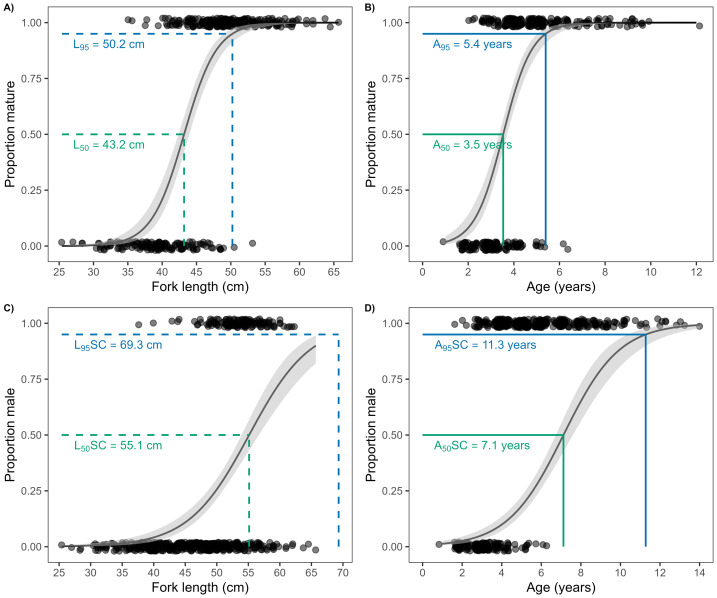
Female size at maturity (A), female age at maturity (B), size at sex change (C), and age at sex change (D) for *L. olivaceus* sampled in Palau in 2017–2019 and 2022–2025. Green dashed lines = size at 50% maturity/sex change, blue dashed lines = size at 95% maturity/sex change, green solid lines = age at 50% maturity/sex change, and blue solid lines = age at 95% maturity/sex change. Solid lines represent best-fit models and grey ribbons show 95% confidence intervals.

Female *L*_50_ was estimated to be 43.2 (95% CI [42.5–44.0]) cm and *L*_95_ was 50.2 (48.7–51.7) cm (Fig. 7A and Table 1). Out of all the individuals sampled, 27.7% were below the female *L*_50_ estimated during this study (Fig. 1). Female *A*_50_ was estimated to be 3.5 (3.4–3.7) years and *A*_95_was 5.4 (4.9–5.9) years (Fig. 7B). Size at 50% sex change (*L*_50_*SC*) was estimated to be 55.1 (53.8–56.9) cm and (*L*_95_*SC*) was 69.3 (65.2–74.4) cm (Fig. 7C). Age at 50% sex change (*A*_50_*SC*) was estimated to be 7.1 (6.7–7.7) years and (*A*_95_*SC*) was 11.3 (10.1 -12.7) years (Fig. 7D).

### Mortality

**Figure 8:**
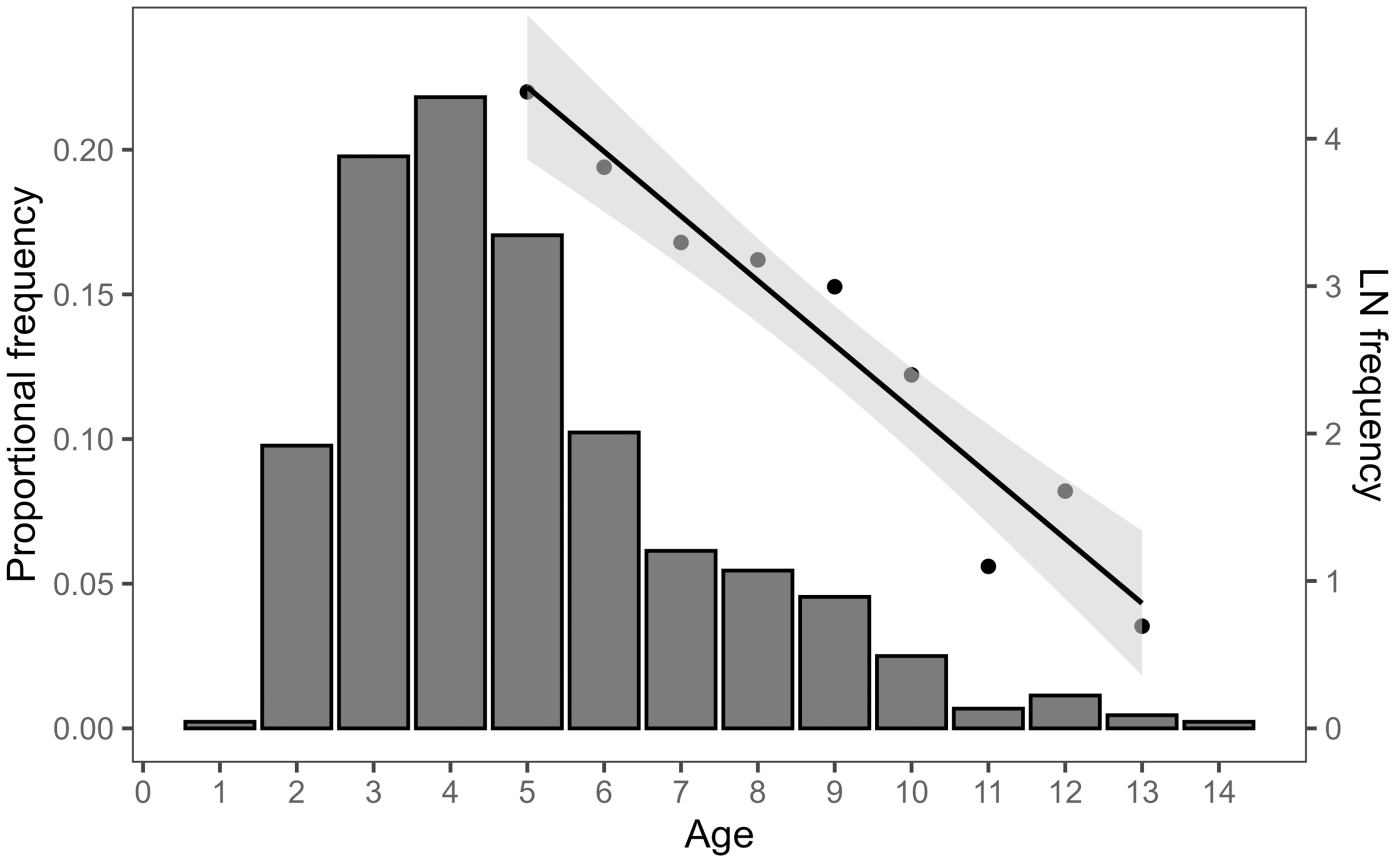
Linearized catch curve for *L. olivaceus* in Palau including data from 2017–2019 and 2022–2025.

Total mortality (*Z*), including 95% confidence intervals, was estimated to be 0.438 (0.335–0.541) year^−1^ using combined 2017–2019 and 2022–2025 catch-at-age data (Table 1). The fully recruited age was estimated to be 5 years, and the catch-curve regression was fitted to age classes ≥ 5 years (Fig. 8). Log-transformed abundance declined linearly with age across the fully recruited ages (*R*^2^ = 0.935, *p* < 0.001). Natural mortality (*M*) estimates were similar across all three methods with a mean value of 0.358 ± 0.05 year^−1^ (Table 1). Fishing mortality (*F*) was estimated to be 0.08 year^−1^, with a low ratio of fishing mortality to natural mortality (*F/M*) of 0.22 (Table 1).

## Discussion

This study estimated age, growth, and reproductive traits of the longnose emperor, *L. olivaceus*, in Palau. *L. olivaceus* was found to have a moderate lifespan, growth rate, and maturity size/age. We found that in Palau the species reaches smaller sizes, grows at a faster rate, and has a shorter lifespan compared to other locations at higher latitudes, including Japan, Australia, and French Polynesia. Our study provides evidence that *L. olivaceus* spawns throughout the year and exhibits functional protogynous hermaphrodism. In combination, this new information can be used to parameterise more accurate stock assessments and inform management decisions for this commercially important species in the future.

### Life-history parameters

The current study used data collected by Prince et al. (2022) together with additional data to provide an updated size at maturity estimate for *L. olivaceus* in Palau using histological methods. A slightly higher female L_50_ value of 43.2 cm FL was estimated compared to the previous estimate by Prince et al. (2022) of 42.3 cm FL. This value is also higher than previous macroscopic estimates from the northern reefs of Palau of 40.9 cm FL (Prince et al., 2015) and 40.5 cm FL (Prince, 2016). These differences could be caused by regional variations in fish populations within Palau, with the northern reefs known to have lower fishing pressure and higher reef fish biomass compared to southern reefs (Muller-Karanassos et al., 2021). In addition, macroscopic techniques have been found to significantly overestimate the proportion of mature females (Vitale, Svedäng & Cardinale, 2006), which may explain the lower estimates found using macroscopic methods compared to histological methods. In French Polynesia, L_50_ for females was found to be much lower than in Palau (38 cm TL; 34.4 cm FL) (Filous et al., 2022), despite a similar age at maturity in both locations (3 years in French Polynesia and 3.5 years in this study). Furthermore, sex change was estimated to occur at 45 cm TL (40.9 cm FL) and 4 years in French Polynesia (Filous et al., 2022), which is much lower than the estimates from this study (55.1 cm FL and 7.1 years). However, these values are likely underestimates since Filous et al. (2022) used macroscopic methods and estimated size and age at sex change based on the initial appearance of transitional females rather than the size or age at which 50% of individuals had transitioned to males, as in the current study.

Life-history traits including maximum size, maximum age, asymptotic length, and growth rate can be strongly influenced by temperature and latitude, with fish known to grow larger, live longer, and grow faster at lower temperatures and higher latitudes (Cappo, Marriott & Newman, 2013; Taylor et al., 2021). This pattern is consistent with the temperature-size rule, which predicts that ectotherms growing in cooler environments achieve larger adult body sizes due to temperature-dependent effects on metabolic rate, growth efficiency, and the balance between somatic growth and maturation (Atkinson, 1994; Angilletta Jr, Steury & Sears, 2004). In Palau, *L. olivaceus* reached a maximum fork length of 65.7 cm, a maximum age of 14 years, an asymptotic length of 56.4 cm, and a growth rate of 0.443. These values are generally smaller and indicate faster growth than populations at higher latitudes. Maximum sizes at other study sites ranged from 71.7 cm FL in Guam (Kamikawa et al., 2015) to 76.8 cm FL in Japan (Shimose, 2021), and asymptotic lengths were also higher at all study sites (66–69.9 cm FL). Maximum age in Palau was the same as in French Polynesia (Filous et al., 2022) and similar to Australia (15 years) (Currey et al., 2013), but much lower than Japan (22 years) (Shimose, 2021). Growth rates in Palau were higher than in Japan (*K* = 0.333) and French Polynesia (*K* = 0.258), but similar to Australia (*K* = 0.47). This trend largely follows the expected latitudinal pattern of faster growth and smaller size at lower latitudes, likely driven by higher annual temperatures in Palau. However, despite being located at a higher latitude than Palau, fish in French Polynesia matured earlier, changed sex at a younger age, and reached the same maximum age as Palau, despite having a slower growth rate and reaching larger sizes. Similarly, natural mortality was comparable between the two regions (*M* = 0.309 in French Polynesia and *M* = 0.358 in Palau) because maximum age was the same, further suggesting that mortality does not follow the expected latitudinal pattern.

Recent studies have shown that although temperature and latitude can affect growth, size, age, and maturity, there are other factors such as resource availability, habitat type, and competition that can also influence life-history traits between geographic locations. For example, a study conducted in the Great Barrier Reef on *Centropyge bispinosa* (two-spined angelfish) found no predictable latitudinal variation in mortality rates, growth rates, asymptotic or maximum length (Lowe et al., 2021). Rather, local environmental conditions, such as reef type (continental *vs.* oceanic), seemed to be more important in predicting life-history traits (Lowe et al., 2021). Another study found that even though wrasses in the Philippines matured sooner and had shorter lifespans compared to wrasses in the Great Barrier Reef, as was expected, they also grew to large sizes (Lowe et al., 2022). This was assumed to be caused by the higher resource availability in the Philippines, which may offset temperature-driven constraints (Lowe et al., 2022). In addition to these environmental drivers, body-size responses to temperature can also be affected by fishing pressure (Audzijonyte et al., 2020). Higher fishing pressure can directly select for reduced size and age at maturity, and fisheries-induced evolution may favour smaller, earlier-maturing individuals when large fish are preferentially removed (Jørgensen & Fiksen, 2010). Therefore, differences in local environmental conditions, resource availability, and/or fishing pressure may explain the different life-history traits observed in French Polynesia compared to Palau.

Total mortality of *L. olivaceus* in Palau, including data from both time periods, was higher (0.438 year^−1^) compared to the Great Barrier Reef (0.28 year^−1^) (Currey et al., 2013), which may indicate fishing pressure is higher in Palau. However, fishing mortality estimated in this study was low (0.08 year^−1^), accounting for only approximately 18% of total mortality. A recent assessment of *L. olivaceus* stocks in Palau using the length-based spawning potential ratio (LBSPR) method revealed a low spawning potential ratio of 29% and high *F/M* of 1.73 (Muller Karanassos et al., in press). This is much higher than the estimated *F/M* in this study of only 0.22. This discrepancy may reflect strong size-selective fishing on individuals prior to full recruitment, as indicated by the high proportion of immature fish in the catch (27.7%), which is captured by LBSPR but not by catch-curve methods that rely on fully recruited age classes. Importantly, LBSPR relies heavily on accurate local life-history parameters and can be highly sensitive to these inputs; therefore, the updated estimates provided here will reduce uncertainty in stock status estimates and strengthen the reliability of LBSPR assessments for Palau.

Morphometric relationships for this species were broadly consistent with previously published estimates. The length-length relationship (FL to TL) closely matched values reported from French Polynesia (Filous et al., 2022) where the intercept and slope were estimated as *a* = 2.34 and *b* = 1.04, compared to *a* = 1.886 and *b* = 1.021 in the present study. Similarly, the length–weight relationship was comparable to estimates from Japan, where *a* = 0.0161 and *b* = 2.978 (Shimose, 2021), *versus a* = 0.0158 and *b* = 2.955 here. In contrast, parameters from Guam (*a* = 0.02, *b* = 2.93) (Kamikawa et al., 2015) and New Caledonia (*a* = 0.0294, *b* = 2.93) (Kulbicki, Guillemot & Amand, 2005) differed slightly from our results. Although these differences are relatively small, they can lead to substantial variation in estimated biomass, underscoring the importance of using locally-derived morphometric relationships when converting length to weight.

### Spawning season and sexuality

There was evidence that *L. olivaceus* spawns in several months of the year including March to June, August, September, November, and December. Similarly, a recent study investigating an *L. olivaceus* spawning aggregation site in Palau, found fish aggregate every month of the year around the new moon, with fish density highest from February to May, and decreasing during the summer months (June to August), with a second smaller peak in September and October (Muller Karanassos et al., 2026). Spawning duration of reef fish has previously been linked to seawater temperature, with shorter spawning periods occurring in lower temperature regions compared to higher temperature regions (Shimose & Nanami, 2015; Zarco-Perello et al., 2022). Annual seawater temperature in Palau is high, ranging from ∼28.5–30 °C, which may explain why *L. olivaceus* was found to spawn in numerous months across the year. Similarly, in Rangiroa, French Polynesia, *L. olivaceus* was also found to have an extended spawning season, with peak activity occurring during the austral spring, summer, and fall, and a decrease during winter months (Filous et al., 2022). Conversely, temperatures in Okinawa, Japan, and the Great Barrier Reef, Australia are generally lower and have a larger range (∼21–30 °C and ∼24–30 °C respectively), where *L. olivaceus* was found to have more restricted spawning seasons (April to June in Okinawa and September to October in the Great Barrier Reef) (Currey et al., 2013; Shimose, 2021).

Numerous studies have found evidence of sex change in lethrinid species, including both functional protogynous hermaphroditism (mature females transitioning to males) (*e.g.*, Ebisawa, 1997; Ebisawa, 2006; Sadovy, Mitcheson & Liu, 2008; Taylor et al., 2018) and non-functional protogynous hermaphroditism (immature fish containing both ovarian and testicular tissues) (*e.g.*, Grandcourt et al., 2010; Currey et al., 2013; Taylor, Oyafuso & Trianni, 2017). In the current study, male *L. olivaceus* were found to be larger and older than females, which was previously observed by Filous et al. (2022) and Shimose (2021). In addition, no immature males were observed and no males below 4 years old were found. Four hermaphrodites, ranging in size from 48.5–50.4 cm, containing both mature female and developing male reproductive tissues, were identified. Shimose (2021) also found evidence of sex change in adult *L. olivaceus* with gonads containing spermatozoa and residual oocytes. These observations indicate that this species is a functional protogynous hermaphrodite, where fish mature and reproduce as a female first and then transition to a male. Fish species that change sex during their lifetime are particularly vulnerable to fishing pressure because the selective removal of larger individuals often results in disproportionately removing males, leading to skewed sex ratios (Alonzo & Mangel, 2004). In the current study, there were almost twice as many mature females (*n* = 246) than males (*n* = 131) observed, which could reflect natural population structure or potentially result from selective removal of larger males. However, no direct data on fishing effort or gear selectivity are available to determine whether fishing contributed to this pattern.

### Limitations and management implications

Although locally derived life-history information is essential for data-poor assessments of coral reef fishes, some parameters remain difficult to estimate accurately. For example, if a population has already been impacted by fishing as it has in this case, estimates of *L*_∞_ may be biased downward because the largest and oldest individuals have likely been removed, truncating the sampled size distribution and biasing growth curves fit to available data (*e.g.*, Bolser et al., 2018). Likewise, natural mortality (*M*) is notoriously challenging to estimate reliably because it must usually be inferred indirectly, is confounded with other life-history and fishery parameters, and carries high uncertainty in stock assessments (Maunder et al., 2023). In contrast, size at maturity estimates can be relatively robust to fishing-induced truncation, provided the sampled size range includes a sufficient number of immature and mature individuals to fit a maturity ogive. In such cases, the absence of very large fish may have limited influence because they contribute little additional information once maturity is already near 100%. However, if size truncation is severe and the removed large individuals have systematically different maturity schedules (*e.g.*, higher *L*_50_), then fishing could bias maturity estimates. Therefore, while local gonad-based maturity data are valuable, their reliability should be interpreted considering potential sampling truncation and selective removal. In heavily fished locations, it may therefore be more appropriate to draw on rigorously vetted databases—such as the one developed by Prince, Wilcox & Hall (2023)—which provide family-level *L*_50_*/L*_∞_ and *M/K* ratios that may be more robust than locally estimated individual parameters. The life-history parameters estimated in this study could be compared with, and potentially contribute to, databases of this kind.

A new coastal fisheries management plan developed for the state of Koror, Palau, in 2021, proposes a minimum size limit of 18 inches (45.7 cm) for *L. olivaceus,* although this regulation has not yet been implemented. This limit was determined based on local fisher knowledge together with the *L*_50_ estimates reported by Prince et al. (2015) and Prince (2016). However, these estimates were derived from macroscopic assessments of gonad maturity from the northern reefs of Palau. The present study provides a more robust estimate of size at maturity using histological methods and samples collected from the state of Koror, thereby providing improved information for determining appropriate minimum size limits. To meet the rule of thumb for minimum size limits developed by Prince & Hordyk (2018) of *L*_50_ × 1.1 or 1.2, the value currently proposed for *L. olivaceus* would need to be increased to at least 18.7 inches (47.5 cm) to protect the spawning potential of this species. Furthermore, a maximum size limit could also be implemented to protect males in addition to females, as harvest slot limits have previously been shown to protect large, reproductively important individuals (Gwinn et al., 2015). An appropriate maximum size limit could be based on the size at 50% sex change (55.1 cm), which would allow males above the transition size to contribute to reproduction (Alonzo & Mangel, 2004). Additional management options could include seasonal or lunar closures around the new moon, in addition to protection of known spawning aggregation sites (Muller Karanassos et al., 2026).

## Conclusions

This study provides a comprehensive assessment of age, growth, and reproductive characteristics of *L. olivaceus* in Palau. While previous work has estimated size at maturity for this species in Palau, this study is the first to estimate age and growth parameters and integrate these with reproductive analyses. By combining otolith-based age determination with histological assessment of maturity and spawning patterns, this study provides new life-history information for a commercially important species at lower latitudes. Our results show that *L. olivaceus* in Palau exhibits relatively rapid growth, smaller sizes, and a shorter lifespan compared with populations from higher-latitude regions, underscoring pronounced geographic variation in life-history traits. Evidence of year-round spawning and functional protogynous hermaphroditism highlights potential vulnerability to fishing pressure, particularly where individuals may be selectively targeted during spawning aggregations. In this context, the life-history parameters and size-related estimates presented here provide a critical foundation for evaluating population status, parameterising robust stock assessments, and informing effective, location-specific fisheries management strategies for *L. olivaceus* in Palau and similar tropical systems. Management approaches such as size-based harvest regulations and protection of spawning aggregations may be particularly important for maintaining sustainable populations of this species. Future research should focus on quantifying fishing pressure and monitoring population trends to further support effective management of this species in Palau.

##  Supplemental Information

10.7717/peerj.21247/supp-1 Supplemental Information 1 Sex-combined length-length (*n* = 448) (A) and length-weight (*n* = 526) (B) relationships for *L. olivaceus* sampled in Palau.

10.7717/peerj.21247/supp-2 Supplemental Information 2 Gonadosomatic index (GSI) of mature female and male *L. olivaceus* sampled in Palau across months and lunar day (0 = new moon)

10.7717/peerj.21247/supp-3 Supplemental Information 3 Growth parameters of linear length-length (*n* = 448) and nonlinear length-weight (*n* = 526) relationships for sex-combined *L. olivaceus* sampled in Palau, with 95% confidence intervals FL, fork length (cm), TL, total length (cm), W, total weight (g).

10.7717/peerj.21247/supp-4 Supplemental Information 4 Life history parameters of *L. olivaceus* across study locations

10.7717/peerj.21247/supp-5 Supplemental Information 5 Longnose emperor biological sampling

10.7717/peerj.21247/supp-6 Supplemental Information 6 Longnose life history parameters comparison

10.7717/peerj.21247/supp-7 Supplemental Information 7 Longnose emperor life history analysis code

## Additional Information and Declarations

### Data Availability

The following information was supplied regarding data availability: The raw data and code are available in the Supplemental Files.
