# Supplementary figures and images for: Life-history and reproductive traits of a key coral reef fishery species: the longnose emperor (Lethrinus olivaceus) in Palau
Source: PeerJ. 2026 May 7;14:e21247. doi: 10.7717/peerj.21247 (PMC13157811; doi:10.7717/peerj.21247)

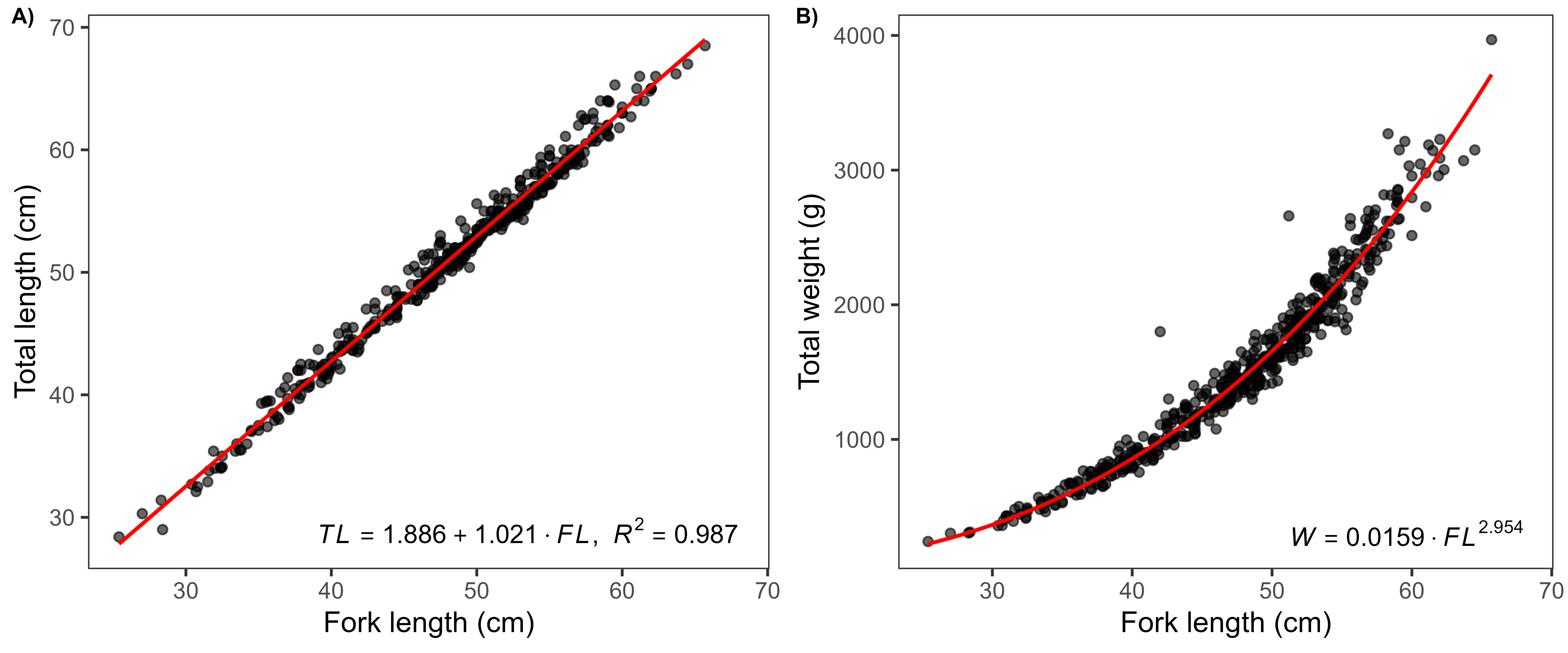

Supplement: Supplemental Information 1 — (A) and length-weight (n = 526) (B) relationships for L. olivaceus sampled in Palau. [file peerj-14-21247-s001.png]

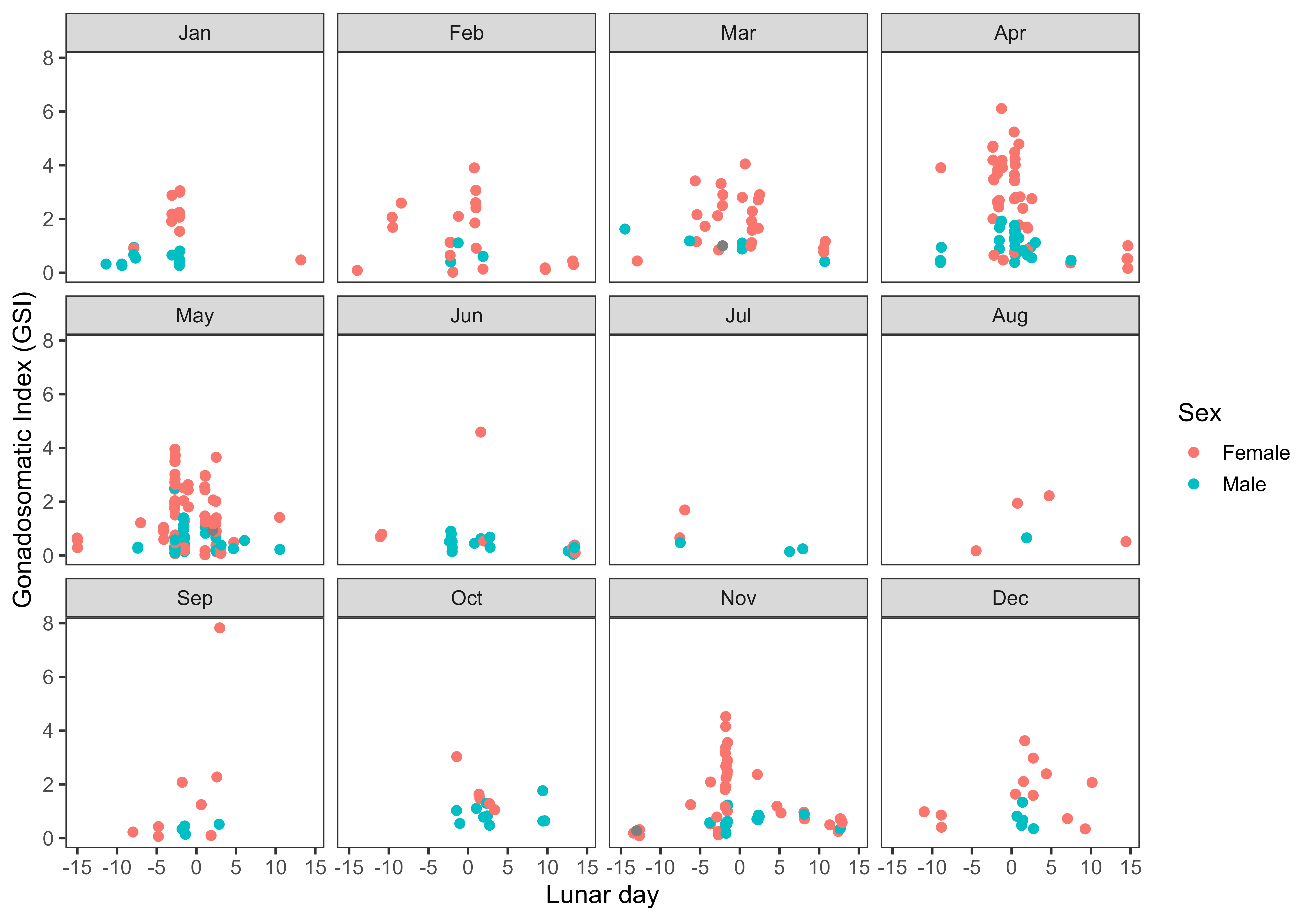

Supplement: Supplemental Information 2 [file peerj-14-21247-s002.png]
