# Supplementary material for: Life-history and reproductive traits of a key coral reef fishery species: the longnose emperor (Lethrinus olivaceus) in Palau
Source: PeerJ. 2026 May 7;14:e21247. doi: 10.7717/peerj.21247 (PMC13157811; doi:10.7717/peerj.21247)
Supplement: Supplemental Information 3 — FL, fork length (cm), TL, total length (cm), W, total weight (g). [file peerj-14-21247-s003.docx]

**Table S1.** Growth parameters of linear length-length (n = 448) and nonlinear length-weight (n = 526) relationships for sex-combined *L. olivaceus* sampled in Palau, with 95% confidence intervals. FL = fork length (cm), TL = total length (cm), W = total weight (g).

| **X** | **Y** | **a** | **b** | **R^2^** | **p-value** |
| --- | --- | --- | --- | --- | --- |
| FL | TL | 1.886 (1.351 - 2.421) | 1.021 (1.010 - 1.032) | 0.987 | <0.001 |
| FL | W | 0.0158 (0.0120 – 0.0205) | 2.955 (2.888 – 3.024) | - | <0.001 |
