# Supplementary material for: Life-history and reproductive traits of a key coral reef fishery species: the longnose emperor (Lethrinus olivaceus) in Palau
Source: PeerJ. 2026 May 7;14:e21247. doi: 10.7717/peerj.21247 (PMC13157811; doi:10.7717/peerj.21247)
Supplement: Supplemental Information 4 [file peerj-14-21247-s004.docx]

**Table S2.** Life history parameters of *L. olivaceus* across study locations.

| **Life history parameter** | **Palau** | **French Polynesia** | **Japan** | **Australia** | **Guam** | **New Caledonia** |
| --- | --- | --- | --- | --- | --- | --- |
| *L_max_* (cm) | 65.7 | 73.4 | 76.8 | 73.3 | 71.7 | 72.5 |
| *A_max_* (years) | 14 | 14 | 22 | 15 | - | - |
| *L_∞_* constrained (cm) | 56.4 | 68.2 | 69.9 | 66 | - | - |
| *K* constrained (year¯¹) | 0.443 | 0.258 | 0.333 | 0.47 | - | - |
| *L_∞_* unconstrained (cm) | 57.2 | 73.4 | 71.9 | - | - | - |
| *K* unconstrained (year¯¹) | 0.387 | 0.18 | 0.258 | - | - | - |
| *t_0_* unconstrained (years) | -0.324 | -0.82 | -0.613 | - | - | - |
| Female *L_50_* (cm) | 43.2 | 34.4 | - | - | - | - |
| Female *L_95_* (cm) | 50.2 | 42.7 | - | - | - | - |
| Female *A_50_* (years) | 3.5 | 3 | - | - | - | - |
| Female *A_95_* (years) | 5.4 | 4 | - | - | - | - |
| *L_50_SC* (cm) | 55.1 | 40.9 | - | - | - | - |
| *L_95_SC* (cm) | 69.3 | - | - | - | - | - |
| *A_50_SC* (years) | 7.1 | 4 | - | - | - | - |
| *A_95_SC* (years) | 11.3 | - | - | - | - | - |
| Source | This study | Filous et al. (2022) | Shimose (2021) | Currey et al. (2014) | Kamikawa et al. (2015) | Kulbicki et al. (2005) |

Note: *L_max_* = maximum length, *A_max_* = maximum age, *L_∞_* = mean asymptotic length, *K* = von Bertalanffy growth coefficient, *t_0_* = age at which fork length equals zero, *L_50_* = size at 50% maturity, *L_95_* = size at 95% maturity, *A_50_* = age at 50% maturity, *A_95_* = age at 95% maturity, *L_50_SC* = size at 50% sex change, *L_95_SC* = size at 95% sex change, *A_50_SC* = age at 50% sex change, *A_95_SC* = age at 95% sex change. Length estimates are in fork length (cm).
